# Vascular Dementia and Risk Factors in Ethiopia

**DOI:** 10.1002/alz70857_098872

**Published:** 2025-12-24

**Authors:** Yared Z Zewde

**Affiliations:** ^1^ College of Health Sciences, Addis Ababa University, Addis Ababa, Ethiopia; Global Brain Health Institute, San Francisco, CA, USA

## Abstract

**Background:**

Vascular dementia (VaD) is the second most common form of dementia globally, resulting from impaired blood flow to the brain. In Ethiopia, where non‐communicable diseases are rising, understanding the prevalence and risk factors of VaD is critical for public health planning. However, limited research exists on VaD in this low‐resource setting. This study aimed to assess the prevalence of VaD and identify associated risk factors among adults in Ethiopia.

**Method:**

A hospital‐based cross‐sectional study was conducted at Lancet General Hospital, a multispecialty private center located in Addis Ababa, Ethiopia, from January 2023 to December 2024. Adults aged 50 years and older who presented with memory, cognitive and behavioral complaints were enrolled consecutively. Data were collected through structured interviews, clinical assessments, and cognitive screening using the Mini‐Mental State Examination (MMSE). Vascular dementia was diagnosed based on DSM‐5 criteria. Risk factors, including hypertension, diabetes, dyslipidemia, and cardiovascular disease, were assessed using standardized tools. Logistic regression analysis was performed to identify independent risk factors for VaD.

**Result:**

A total of 155 dementia patients were enrolled. The median age was 70 years and it ranges from 50‐92 years. Males accounted 56.2%. The prevalence of VaD was found to be 71%. Hypertension (OR = 2.3, 95% CI: 1.7–3.1), diabetes (OR = 1.9, 95% CI: 1.3–2.8), and dyslipidemia (OR = 1.7, 95% CI: 1.2–2.4) were significantly associated with VaD. Older age and low educational attainment were also independently associated with increased risk of VaD.

**Conclusion:**

Vascular dementia is a significant public health issue in Ethiopia, particularly among older adults with cardiovascular risk factors and low literacy. These findings underscore the importance of targeted interventions to control modifiable risk factors, such as physical activity and proper management of hypertension, diabetes, and dyslipidemia. Public health strategies which promote healthy lifestyle and adult education are important to reduce the growing burden of dementia in Ethiopia.

